# Monitoring motor capacity changes of children during rehabilitation using body-worn sensors

**DOI:** 10.1186/1743-0003-10-83

**Published:** 2013-07-30

**Authors:** Christina Strohrmann, Rob Labruyère, Corinna N Gerber, Hubertus J van Hedel, Bert Arnrich, Gerhard Tröster

**Affiliations:** 1 Wearable Computing Lab.,, ETH Zurich, Gloriastrasse 35, Zurich, Switzerland; 2 Pediatric Rehab Research Group, Rehabilitation Center, University Children’s Hospital Zurich, Mühlebergstrasse 104, Affoltern am Albis, Switzerland

**Keywords:** Experiments, Signal processing, Rehabilitation, Children, Wearable sensors, Motor performance

## Abstract

**Background:**

Rehabilitation services use outcome measures to track motor performance of their patients over time. State-of-the-art approaches use mainly patients’ feedback and experts’ observations for this purpose. We aim at continuously monitoring children in daily life and assessing normal activities to close the gap between movements done as instructed by caregivers and natural movements during daily life. To investigate the applicability of body-worn sensors for motor assessment in children, we investigated changes in movement capacity during defined motor tasks longitudinally.

**Methods:**

We performed a longitudinal study over four weeks with 4 children (2 girls; 2 diagnosed with Cerebral Palsy and 2 with stroke, on average 10.5 years old) undergoing rehabilitation. Every week, the children performed 10 predefined motor tasks. Capacity in terms of quality and quantity was assessed by experts and movement was monitored using 10 ETH Orientation Sensors (ETHOS), a small and unobtrusive inertial measurement unit. Features such as smoothness of movement were calculated from the sensor data and a regression was used to estimate the capacity from the features and their relation to clinical data. Therefore, the target and features were normalized to range from 0 to 1.

**Results:**

We achieved a mean RMS-error of 0.15 and a mean correlation value of 0.86 (*p*<0.05 for all tasks) between our regression estimate of motor task capacity and experts’ ratings across all tasks. We identified the most important features and were able to reduce the sensor setup from 10 to 3 sensors. We investigated features that provided a good estimate of the motor capacity independently of the task performed, e.g. smoothness of the movement.

**Conclusions:**

We found that children’s task capacity can be assessed from wearable sensors and that some of the calculated features provide a good estimate of movement capacity over different tasks. This indicates the potential of using the sensors in daily life, when little or no information on the task performed is available. For the assessment, the use of three sensors on both wrists and the hip suffices. With the developed algorithms, we plan to assess children’s motor performance in daily life with a follow-up study.

## Background

For children with neurological deficits, such as cerebral palsy or stroke, it can be indicated to undergo stationary treatment in specialized rehabilitation facilities to improve specific aspects of their impairments. In contrast to adults, such a stay does not only include time for therapies and care, but also for education. Thus, time is limited and should be used as efficiently as possible. Therapy should address the needs of the individual child as efficiently as possible, adapting to functional improvements as timely as possible. To capture these improvements during rehabilitation, rehabilitation services should apply objective outcome measures regularly during the patient’s stay [1,2]. This might be even more important when treating children as they sometimes are not able to give a feedback as precise as adults are able to or as one has to rely on subjective parental information. To date, various outcome measures such as the Gross Motor Function Measure [3], the Pediatric Evaluation of Disability [4], the Functional Independence Measure for Children (WeeFIM) [5], the Movement Assessment Battery for Children [6] or the 10 Meter Walk Test [7] are used to assess motor capacity or performance of these children. In this manuscript, we refer to “capacity” when a test is performed in a standardized environment according to a clearly defined protocol, while “performance” refers to assessing the functioning of the patient in his/her own environment during daily life situations (see also the International Classification of Functioning, Disability and Health (ICF) at http://www.who.int/classifications/icf/eng). Such tests often rely on the evaluation of standardized tasks or periodically recurring everyday situations. Those are then usually rated by a specialist based on timing or quantity, either directly or from video recordings. It has been shown that these measures provide valuable information for determining whether movement capacity or performance increased during rehabilitation or not and eventually for adapting the individual rehabilitation program to achieve the best possible results [8,9].

However, there might still be a gap between motor capacity during a rehabilitation stay and motor performance during normal daily life activities at home [10]. While the child might improve during rehabilitation sessions, e.g. by using the affected arm more, it might not do that in daily life. Additionally, it might also be, that outcome measures of motor performance, which are often applied in the form of questionnaires [11], might not accurately reflect the actual motor performance of the child in its familiar environment [12]. To address these issues, we would like to assess performance in daily life using wearable sensors. Wearable technology has been shown to have potential for providing quantitative measures of motor function during rehabilitation [13,14]. Indeed, we aim to extract certain parameters from such sensor data, which could inform us accurately about the quality and quantity of movement behavior of the child. Previous research has focused on a one-time assessment rather than a longitudinal assessment over several weeks and most of the studies have been performed looking at lower limb function in adults.

As a first step, we wanted to investigate the feasibility of monitoring upper and lower motor capacity of children undergoing rehabilitation and performed a longitudinal study over a course of 4 weeks to address the following research questions: 

1. How accurately can expert rating of motor capacity of several tasks be estimated from the sensor data?

2. What features^a^ might prove most sensitive to determine changes in motor capacity of children undergoing rehabilitation?

3. How many sensors are needed to assess motor capacity of upper and lower extremities?

4. Can the assessment be generalized across different motor tasks in terms of used features and sensors?

This approach serves to develop and validate our algorithms, which can then serve as a basis for assessing motor performance in unrestricted daily life.

### Related work

Wearable technology has been used by various researchers for automatic assessment of motor function. In this section, we present related work on automatic assessment of motor function from sensor data collected during predefined motor skill assessment tasks, during daily living, and the application of sensor-based assessment on children.

#### ***Motor function assessment using predefined tasks***

In most related work, researchers monitored movement during predefined motor assessment tasks using wearable technology with the goal to assess movement function with either signal processing or machine learning. The ground truth was obtained by experts’ ratings. Using machine learning, the most common approach is to extract features from sensor data, and to perform a feature selection and a classification [13].

With such a machine learning approach motor recovery of stroke survivors has been assessed using accelerometers attached to different positions of the patient’s affected arm [15,16]. Similarly, authors [17,18] extracted statistical features from accelerometer data to predict motor function scores during different tasks. They were able to predict the scores with an error of 10%. In [19], authors describe the automatic assessment of 7 out of the 17 tasks of the Wolf Motor Function Test (WMFT [20]) using an inertial measurement unit (IMU) and an overhead camera. Bento et al. assessed the task completion time of the WMFT automatically from wearable-sensor-based motion capture using an adaptive threshold, which was compared to the manually stopped time [21]. Additionally, they assessed Functional Ability Scores (FAS) using a decision tree classifier. The classification between affected and unaffected arm performing the WMFT using sensor data was demonstrated for a post-stroke patient in [22]. They validated their approach with one subject who was equipped with a single IMU on her wrist. They calculated features from the IMU data which they used for classification using a Naïve Bayes classifier.

Zhang et al. followed a more exploratory approach, aiming towards a temporally fine-grained motor performance assessment [23]. They used Dynamic Time Warping^b^ to compare movement trajectories of the affected and unaffected arm.

Even though the use of accelerometers and IMUs has been shown to provide valuable information, researchers also investigated other modalities such as e.g. a home-based rehabilitation system based on an optical linear encoder, which seemed to be a promising approach being affordable while providing reliable results, which was evaluated against an optical motion capture system [24].

However, the presented approaches using body-worn sensors focused on a one-time assessment of motor performance rather than investigating longitudinal development of motor function during rehabilitation.

#### ***Motor performance assessment in daily living***

To assess the reliability and validity of accelerometry for measuring upper extremity rehabilitation outcome, Uswatte et al. [25] equipped patients with accelerometers during daily life. As ground truth, a semi-structured interview of real-world arm usage was performed. They found that a simple measure of arm usage can be used to assess rehabilitation outcome. Arm activity during three consecutive days was assessed in [26]. They found a significant relation to ratings of motor assessment and wrist acceleration in terms of the ratio of arm usage between affected and unaffected arm. The assessment of gait parameters has been shown to be very useful for motor function assessment and can be extracted from wearable sensor units [27].

#### ***Motor function assessment of children***

It was shown that readings from an accelerometer provide valuable information on walking and daily physical activities of children with hemiplegia [28]. Harms et al. demonstrated the use of accelerometers integrated in a shirt for monitoring posture of children [29]. Pressure-instrumented shoes have been used for the assessment of the severity of toe-walking of children with cerebral palsy (CP) [30,31]. However, to the best of our knowledge longitudinally monitoring motor function of children undergoing stationary rehabilitation treatment in a clinic with body-worn sensors was not investigated to date.

## Methods

### Experiment design

To assess the sensitivity of our sensors to monitor changes in motor function over time, we performed a study on a convenience sample of 4 children that participated in our experiments over a course of 4 weeks while undergoing clinical rehabilitation for at least 4 weeks. In the 2 children who acquired a stroke, the time since lesion was 10 weeks (ID 2) and 12 years (ID 4), respectively. Inclusion criteria were: Neurological diagnosis leading to stationary stay in our rehabilitation center with focus on upper extremity motor function, age 5 years to 18 years, cognitive ability to understand the aim of the tasks defined below. Characteristics of the participants including their rating according to the WeeFIM [5] can be found in Table 1. Before testing, written informed consent was obtained from all children and their parents to participate in the study, and the study was approved by the Ethics Committee of the Canton of Zurich, Switzerland. Written informed consent was also obtained from the parents for publication of both patient data and all accompanying images.

**Table 1:** Details on participating children

**ID**	**Age**	**Height**	**Weight**	**Gender**	**Diagnosis**	**WeeFIM[**[5]**]**	
	**[years]**	**[cm]**	**[kg]**			**Self care**	**Mobility**
1	9.8	130	37	Boy	Cerebral palsy	68%	71%
					(GMFCS level III)		
2	9.0	148	34	Girl	Stroke	79%	91%
					(hemiparesis right)		
3	11.2	144	42	Girl	Cerebral palsy	82%	57%
					(GMFCS level III)		
4	12.0	142	32	Boy	Stroke	98%	63%
					(hemiparesis right)		

Abbreviations: ID = identification; GMFCS = Gross Motor Function Classification System; WeeFIM = Functional Independence Measure for children. The percentage values of the WeeFIM Self Care item (mainly upper extremity dependent) and the Mobility item (mainly lower extremity dependent) reflect the performance in comparison with healthy coeval children.

For our study, each child performed predefined motor tasks once a week. The predefined motor tasks were selected in collaboration with movement scientists and occupational therapists and consisted of items from established and validated motor assessments, namely from the Jebsen-Taylor Hand Function Test [32], the Graded and Redefined Assessment of Strength, Sensibility and Prehension [33], the Nine-Hole Peg Test [34], and the Timed Up and Go Test [35]. Furthermore, 3 additional items were added to better cover playful, locomotor, and wheelchair activities. This resulted in a short (important factor when measuring children) but very comprehensive motor assessment covering many movements performed during ADL tasks. Sessions were video-recorded for labeling of sensor data and additional clinical scoring by experts. In the following subsections, we present the measurement device, the experimental procedure, and details on the performed tasks.

#### ***Measurement device***

**Figure 1:**
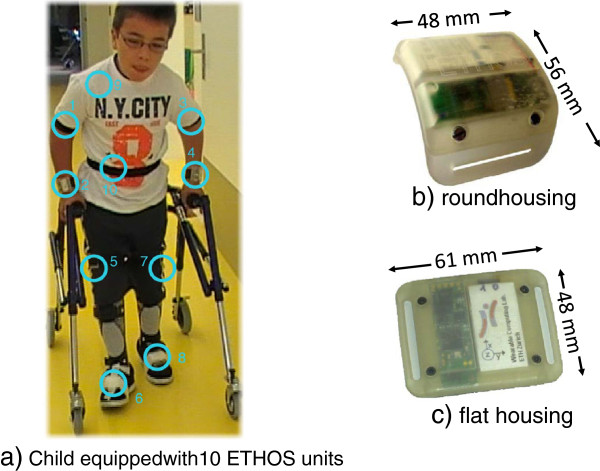
**Child wearing sensors and close ups of the sensors.a)** Child wearing 10 ETHOS units (highlighted with circles; bracelet **(b)** and flat **(c)** housing types were used) during walking with a walking frame.

Our measurement device was the ETH Orientation Sensor (ETHOS), a small and unobtrusive IMU that was developed and applied in previous work [36]**-**[38]. Note that the sensor is not commercially available. ETHOS combines a 3D accelerometer measuring up to 6 g^c^, a 3D gyroscope measuring up to 1200°/s, and a 3D digital compass. Connectivity is provided by an integrated ANT+ module and a USB interface. The elongated design (W × L × H = 14 × 45 × 4 *mm*) is optimized for attachment along human body limbs. We developed flat and bracelet housings (compare Figure 1) that were fixed to the child’s body using elastic velcro straps. The round housing unit weighed 27 g, and the flat housing unit 22 g. Data were sampled at 100 Hz and stored to a local microSD card for subsequent offline analysis. Temporal alignment of simultaneously recorded data was guaranteed by a dedicated hub that synchronized the on-board real time clocks of attached ETHOS units.

#### ***Experimental procedure***

Children participated in our experiment once per week over a course of four weeks. Each experiment session took about 1 h, was included in the children’s personal rehabilitation plan, and was supervised by two experts. Experiment sessions were scheduled on the same day of the week and at the same time of the day, to minimize circadian influences and other influences, e.g. due to the rehabilitation program, fatigue or previous training effects. During these sessions, 10 ETHOS units were attached to upper and lower extremities and the trunk to monitor full body movements. The sensor setup is depicted in Figure 1.

An additional ETHOS unit was synchronized with the other 10 units for automatic label generation during the experiments.

Prior to performing the actual motor function tasks, children were asked to perform a calibration and synchronization pose, which allowed for later synchronization of the sensor data recordings to the video. The pose consisted of lifting the arms to the side and holding that pose for 3 s and then clapping the hands together, which evoked a peak in the acceleration signal that could be used to synchronize the sensor data to the video recording. This pose was repeated after the completion of the motor function tasks to compensate for any possible sensor drift and to investigate possible sensor displacement during the experiment.

For the motor function assessment, children performed a total of 10 tasks under guidance of two caregivers. If a task was performed wrongly, e.g. using the second hand for support, the task was repeated according to the test protocol.

#### ***Task description***

During each weekly session, children performed a total of 10 tasks described below. All attributes that were scored per task are given in brackets (see below for details on scoring).

##### 

**Turn around cards***(grabbing, movement of fingers, force adjustment)* Children were asked to turn around 4 cards using a single hand (task was repeated with the other hand) as fast as possible. This task investigated the children’s capability of grabbing an object and rotating it.

##### 

**Pick up small objects***(grabbing, releasing, movement of fingers, force adjustment, movement of upper arm)* Children needed to pick up 6 small objects (small beans, coins, and paper clips) one after the other and place them into a bin next to them using a single hand. The task was repeated with the other hand and had to be completed as fast as possible. This addressed the children’s fine motor skills.

##### 

**Figure 2:**
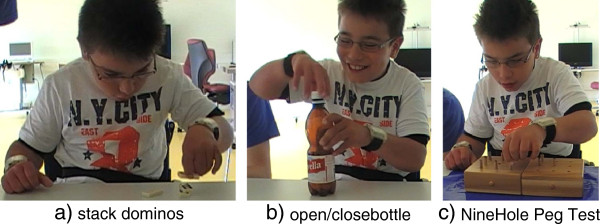
Pictures of tasks performed during weekly sessions.

**Stack dominos***(grabbing, placing, movement of fingers, force adjustment, movement of upper arm)* Four dominos lying next to each other had to be picked up one after the other and put on top of one to build a stack using a single hand as fast as possible. The task was repeated with the second hand. A snapshot of the task is depicted in Figure 2.

##### 

**Lift and replace bigger objects***(stabilizing, placing, force adjustment, movement of upper arm)* Children needed to lift and move 5 bigger bins on a table using both hands as fast as possible.

##### 

**Open and close a bottle***(stabilizing, force adjustment, movement of upper arm, bimanual coordination)* Opening and closing a bottle (of a drink) is a combined bi-manual pro- and supination movement. The task was supposed to be completed as fast as possible and was repeated with both hands. For each trial one hand held the bottle and the other held the cap, see Figure 2. The cap needed to be screwed up, lifted completely from the bottle and screwed down again to close the bottle.

##### 

**Use a key***(grabbing, force adjustment, pronation/supination, aiming)* A very common task in daily life is to put a key in a lock and turn it around to open the door. In this task, the children had to take the key from the table, put it in a lock in front of then, turn the key 360° in one direction and then back to the original position. This task was completed as fast as possible using a single hand and was repeated with the other hand.

##### 

**Nine Hole Peg Test (NHPT)***(grabbing, placing, force adjustment)* This test is used on a regular basis in the children’s hospital for fine motor function assessment. Nine small pegs needed to be grabbed out of the first board and be placed into the nine holes of the second board. After completion, all pegs needed to be put back into the first board. The task had to be completed as fast as possible and was repeated with both hands. A snapshot of the task is depicted in Figure 2.

##### 

**Play Ball***(stabilizing, force adjustment, movement of upper arm, bimanual coordination)* For this task, the child threw a ball to a caregiver and caught it when the caregiver threw it back. This activity includes the use of both arms. Thus, symmetry and regularity of the motion could be assessed during this activity.

##### 

**Timed Up and Go (TUG)***(gait attributes: getting up, sitting down, safety of gait, quality of gait, symmetry of gait, step length/clearance, trunk stability, deviation from path. Wheelchair attributes: Symmetry of arm movements, continuity of arm movements, deviation from path)* For the locomotion assessment, an adapted version of the TUG test [35] was included which consisted of walking 10 m, turning around a tin, and walking back 10 m. Children who were ambulatory with a walker but could not stand up by themselves and often used a wheelchair in daily life performed the TUG with the walker and the test was started and ended in a standing position. Additionally, they performed the test with their wheelchair to cover the 10 m, turn, and roll back. One child was not ambulatory and solely performed the test with the wheelchair.

##### 

**Climbing Stairs***(safety of gait, symmetry of gait, trunk stability)* Besides gait, stair climbing is another locomotion activity that was performed by the children who were able to climb stairs. The task was to climb up and down (separately scored) 2 flights of stairs (9 steps per flight) or as many steps as they managed to accomplish.

#### ***Motor capactiy assessment***

The children’s motor function was extensively assessed by two experts independently after the experiments using the video recordings. Thereby, tasks were rated for different attributes on a scale of 1 to 4, where “1 = normal” and “4 = not able to fulfill the attribute of the task”. Scores 2 and 3 were formulated specifically for each task. In general, however, a score of 2 was given when the attribute of the task was fulfilled, but with reduced dexterity (i.e. it looked somewhat impaired), while a score of 3 indicated that the patient needed considerably longer to fulfill the the attribute of task and/or that he or she used compensatory movement strategies. Attributes for upper extremities were grabbing objects, releasing objects, placing objects, movement of fingers, movement of upper arm, force adjustment, stabilizing, bimanual coordination, pronation/supination, aiming and symmetry of arm movements and continuity of arm movements for wheelchair driving. Attributes for the Timed Up and Go (TUG) and stairs task were getting up, sitting down, safety of gait, quality of gait, symmetry of gait, step length/clearance, trunk stability and deviation from path. This led to a total sumscore range from 79 to 316 points.

When the two raters disagreed on the rating, they reevaluated the movement again watching the video recording together and discussed it until they agreed. The rating of a single session took approximately 40 min per rater and around 5 min for agreement evaluation, totaling in 85 min per session. The single measure one-way random intraclass correlation coefficient over all items and all subjects was 0.94 (95% confidence interval [0.92 to 0.95]).

### Data analysis

For the data analysis accelerometer and gyroscope data were used.

To account for possible sensor displacement within a trial and potential differences in the positioning of the sensors between weeks, we did not use data from the individual axes but calculated the magnitude of gyroscope and acceleration data, which was then used for the data analysis.

We found that sensor data collected at the upper arms did not provide additional information compared to data obtained at the wrists. In fact, when movements were performed with the affected hand, signal amplitudes were already comparatively small at the wrists and almost non-existent in the data collected at the upper arms. For our purpose, it was therefore possible to exclude data from the upper arms, reducing the number of sensors to 8. However, for other purposes (e.g. when 3D reconstructions or orientation of the limbs are needed) these data might be useful.

**Figure 3:**
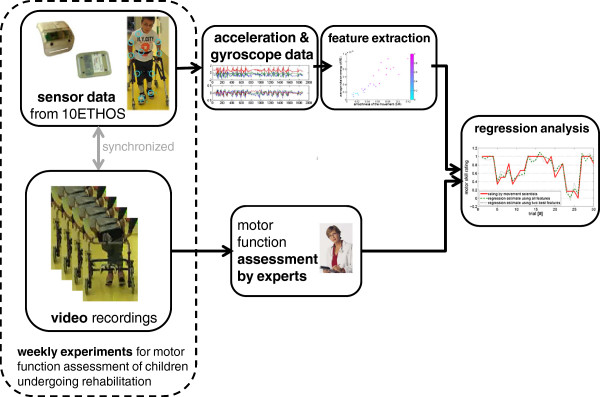
Schematic overview of dataset and procedure of data analysis.

Data were filtered with a low pass filter with a cutoff frequency of 45 Hzk to eliminate noise. Activity labels were generated automatically using data of the labeling sensor. During the experiment, the experiment leader labeled start and end of the task with a fast single and double rotation, respectively, around the labeling sensor’s x-axis. This evoked sharp peaks in the gyroscope data that could be automatically detected. Sensor data and labels were synchronized with video recordings for label validation. A schematic overview of our approach is depicted in Figure 3. For the analysis, the magnitudes of the 3D accelerometer and gyroscope data were calculated and used to account for possible sensor displacement. This will become essential for future assessments, where children will wear sensors during daily life performing unconstrained activities.

#### ***Feature extraction***

To assess motor function development over the course of the 4 weeks, we extracted features from the sensor data of the different tasks. Features were chosen to represent movement characteristics capturing different capacity levels and included promising features from related work [23]. In the following, we describe the extracted features. For each single-handed task, features were calculated from the wrist sensor of the task-performing hand. For the double-handed tasks, features were calculated from the wrist sensors of both hands. Feature vectors for double-handed tasks were thus double the dimension of single-handed tasks. For mobility tasks (wheel chair driving, walking, and stair climbing), features were calculated from the sensor on the hip. To capture asymmetry of movement, we included additional features for these three tasks as described in this section. These additional features were extracted from the sensors on the feet (for level walking), the upper legs (for stair climbing), and the wrists (for wheel chair driving).

Features were calculated for the complete task. The dimension of the feature vector was thus *1 × n*_*features*_ with *n*_*features*_ being the number of features calculated.

##### 

**Figure 4:**
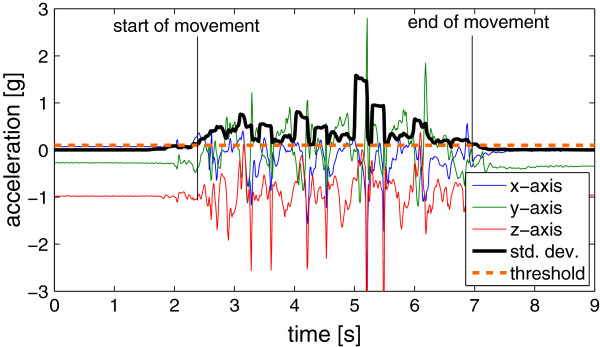
**Acceleration signal of the right wrist during the cards task.** The standard deviation of the acceleration magnitude was calculated with a 0.5 s sliding window with a 0.49 s overlap to estimate when the hand was moved and when not. The detected on- and off-set of movement were then used to estimate task completion time.

**Task completion time (TIME)** The first feature extracted was Task Completion Time since this is to date a quantitative parameter used to characterize motor function [28] that is also commonly used in the clinic. TIME was measured in seconds. We investigated automatic estimation of task completion time from the sensor data. Therefore, the standard deviation of the acceleration magnitude was calculated over a 0.5 s sliding window with a 0.49 s overlap. The onset and the end of movement were detected when this value crossed a threshold as depicted in Figure 4.

Note that this time was not necessarily equivalent to the time measured by movement scientists since they usually started the stopwatch when the object was first touched and stopped it when the last object was released, whereas the sensors only measured whether the hand was moving or not.

##### 

**Mean value of Movement Intensity (MI)** To reflect the intensity of the performed movement, we calculated the mean value of the movement intensity

$$\text{MI}=\bar{\text{MI}(t)}=\frac{1}{T}\overset{T}{\underset{t=0}{\sum }}\text{MI}(t)$$

with $\text{MI}(t)=\sqrt{a_{x}{\left(t\right)}^{2}+a_{y}{\left(t\right)}^{2}+a_{z}{\left(t\right)}^{2}}$ over the complete task, with *a*_*x*_(*t*) (*a*_*y*_(*t*), *a*_*z*_(*t*)) being the acceleration measured on the *x*-axis (*y*-, *z*-axis, respectively) at time *t*. *MI*(*t*) was thus the instantaneous acceleration magnitude at time instance *t*. *MI*(*t*) was measured in g and was independent of the sensor’s orientation, which provided robustness against sensor displacement. Note that MI describes the mean value of the acceleration magnitude over the complete task. We chose the term MI instead of acceleration to avoid any confusion with the 3D time series acceleration.

The mean acceleration is commonly used as a feature in rehabilitation [13] and activity recognition [39]. Clinically, it is related to translational movements in space and can be used to detect postural transitions.

##### 

**Movement Intensity Variation (MIV)** The variation of the movement intensity as defined above indicated the variation of the intensity of the completed task and was intended to indicate the spread of accelerations that were measured while performing the task. It was calculated as follows:

$$\text{MIV}=\frac{1}{T}\overset{T}{\underset{t=0}{\sum }}{\left(\text{MI}(t)-\text{MI}\right)}^{2}$$

with MI being calculated as indicated above and $\text{MI}(t)=\sqrt{a_{x}{\left(t\right)}^{2}+a_{y}{\left(t\right)}^{2}+a_{z}{\left(t\right)}^{2}}$.

MIV can be associated with the spread of acceleration values when looking at a histogram of the acceleration magnitude, as depicted in. Clinically, it is intended to measure the variability of movements.

**Figure 5:**
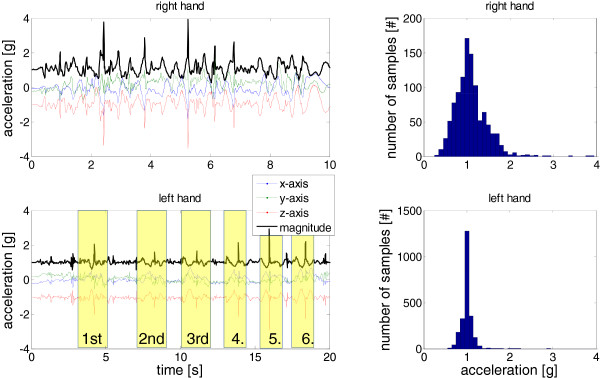
**Acceleration of the right (upper figure) and left (lower figure) wrist of subject 1, week 1, performing the pick up small objects task.** Note that using the left hand, the subject took twice as long to complete the task. While the pick up of the individual 6 objects can be identified for the left hand (indicated with boxes), this is more challenging for the right hand since the movement is faster and includes more dynamic movement as opposed to measuring mainly acceleration due to gravity. The MI and MIV features can be extracted from the histograms depicted on the right side of the figure: MI is indicated with the mean acceleration value and MIV can be associated with the spread of the histogram. The spread and thus MIV are larger for the unaffected (right) hand.

##### 

**Dominant Frequency (DF)** The dominant frequency was calculated as the frequency associated with the highest energy of the Fourier-transformed acceleration signal. The Fourier Transform (FT) of the signal was calculated with a fast Fourier Transform (FFT) algorithm. It reflected the frequency at which the movement was performed. Looking at the frequency at which a movement is performed is well established in related work [13,39]. It provides information about the rate of movement.

##### 

**Figure 6:**
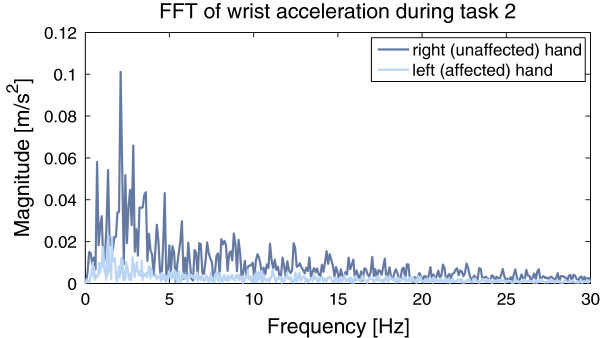
**Energy spectrum obtained via the Fourier Transform of the left (affected) and right (unaffected) hand performing the task “pick up small objects and place in bin” of subject 1.** It can be observed that the energy associated to the dominant frequency of the affected hand was much lower than that of the unaffected hand. We used both the dominant frequency and the energy associated to it as features. The SM parameter of the unaffected side was almost twice as high as that of the affected side.

**Smoothness of Movement (SM)** Common measures of smoothness of movement are jerk measures [40] or spectrum-based measures [41]. It was found that it is important to use a smoothness measure that is dimensionless [40]. Since the children participating in the study performed movements on the affected side rather slowly, we chose to use a spectrum-based smoothness measure over a jerk-based. The smoothness of movement was associated with the energy within the 0.2 Hz bin around the dominant frequency normalized by the entire energy. We used a 0.2 Hz bin to account for the different bin sizes of different signals since the signal duration influences the resolution of the FFT spectrum. Figure 6 exemplarily depicts the FFT spectra of the right and left hand of subject 1 performing task 2 (collect 6 small items and put into bin). The movement of the right hand was smoother compared to the left, which was the subject’s affected side^d^. Energy of certain frequency bands was found to be a powerful feature in the analysis of acceleration data [39]. The smoothness of movement provides information about the periodicity of a movement and offers the possibility to analyze a qualitative aspect of movement that otherwise is seldomly looked at.

##### 

**Average Rotation Energy (ARE)** The average rotation energy was calculated by the mean value of the rotation energy measured by the gyroscope over the whole task and provides information about rotational components of the movement.

##### 

**Range of Angular Velocity (RANG)** The range of angular velocity was calculated by subtracting the minimum value from the maximal value of the angular velocity magnitude and indicated how fast movements were performed.

##### 

**Synchrony of Arm Movement (ArmSync)** We measured synchrony of arm movement for the wheel chair driving task solely. For the data analysis the part of driving straight (10 m towards the tin and 10 m back, not the turning part) was used. Therefore the task was automatically segmented in driving straight and turning using a Sliding Window And Bottom-Up (SWAB) segmentation algorithm [42].

**Figure 7:**
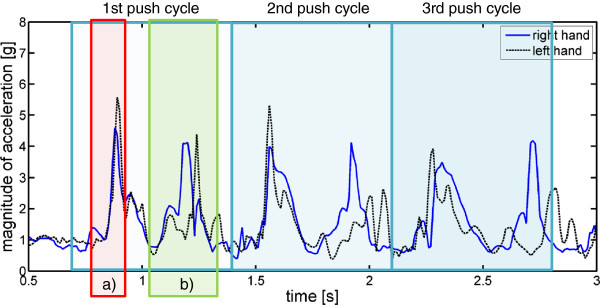
**Right (solid) and left (dashed) wrist acceleration during wheel chair driving of subject 1.** Push cycles are marked. Each push cycle consists of a forward pushing phase **(a)** and a phase when the hands are moved back to prepare for the next push **(b)**. It can be observed that synchrony is high for the forward push **(a)** and lower for the backward release **(b)**. The left hand moves back later, which is the affected hand of this subject.

Figure 7 depicts the magnitude of the acceleration of both wrists of subject 1 during this task.

We measured synchrony of the arm movement during the forward push and the backward release phase by calculating the time difference between the peaks of maximum acceleration of these phases. This time difference was then normalized to cycle duration. From Figure 7 it can be seen that the forward push was performed more symmetric than the backwards release phase. This feature can be used to analyze coordination between arms during wheel chair driving.

##### 

**Gait parameters** Gait parameters such as step and stance duration and symmetry between left and right were found to be an important parameter for gait analysis [43] and were assessed using the sensors on the feet. Steps of each foot were detected using the feet’s acceleration. Each heel strike evoked a high peak in the acceleration magnitude. After a heel strike, the foot is on the ground, yielding an acceleration magnitude close to 1 g ($\hat{=}$ gravity). When the foot is lifted off the ground and swung forwards, the acceleration increases again, which we detected with a threshold. Stance duration was then calculated as the time difference between these two events. We included the average stance and step durations of the trial and the ratio of right to left stance and step durations, adding 4 additional features for the TUG test and the stair climbing test. Step duration was measured as the time between two subsequent heel strikes of the same foot.

**Figure 8:**
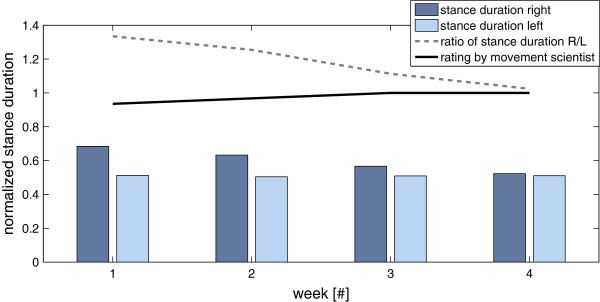
**Calculated stance duration of the left and right foot during the TUG test across the four weeks of subject 2.** It can be seen that the rating increased while the ratio of left to right stance duration approximated 1, the movement thus became less asymmetric.

Figure 8 depicts the calculated stance durations of the left and right foot during the TUG test across the four weeks of subject 2. When calculating the ratio of these two parameters, it seemed that this correlated negatively with the experts’ rating, as expected, see Figure 8. Note that in the figure the experts’ rating was normalized to range between 0 and 1.

#### ***Dataset***

We collected a dataset of 4 subjects participating in our experiments over the 4 weeks. We experienced data loss of a wrist sensor during one experimental session due to low battery and discarded all collected data from this session (subject 3, week 3). The total dataset thus consisted of 15 (4+4+3+4) recording sessions. The sensor positions of which features were calculated and the according dimension of the feature vector and number of data samples per task are summarized in Table 2.

**Table 2:** Overview of calculated features and number of data recordings of the different tasks

**Task**	**Features and sensors**	**# of experiment sessions**
Turn around cards		
Pick up small objects	TIME, MI, MIV, DF, SM,	
Stack dominos	ARE, RANG per task	30 (right and left of
Open and close a bottle	calculated per task from	4 weeks of the subjects
Use a key	wrist of performing hand	- 1 week data loss of subject 3)
NHPT	($\hat{=}$ 7 features)	
Lift and replace bigger objects	TIME, MI, MIV, DF, SM, ARE, RANG	
Play ball	calculated from both (left and	15 (trials of 4 weeks of the subjects
	right) wrists ($\hat{=}$ 14 features)	- 1 week data loss of subject 3)
	TIME, MI, MIV, DF, SM	
	ARE, RANG calculated	
TUG	from hip sensor + average	11 (3 subjects, 4 weeks -
	stance and step duration	1 week data loss)
	and respective ratios	
	between left/right stance	
Stair climbing	and step durations	4 (1 subject, 4 weeks)
	calculated from feet	
	sensors ($\hat{=}$ 11 features)	
TUG (wheel chair)	TIME, MI, MIV, DF, SM, ARE, RANG calculated from the hip + ArmSync calculated from both wrists ($\hat{=}$ 8 features)	11 (3 subjects, 4 weeks - 1 week data loss of subject 3)

Abbreviations: NHPT = Nine Hole Peg Test; TUG = Timed Up And Go; TIME = Task Completion Time; MI = Mean value of Movement Intensity; MIV = Movement Intensity Variation; DF = Dominant Frequency; SM = Smoothness of Movement; ARE = Average Rotation Energy; RANG = Range of ANGular Velocity; ArmSync = Synchrony of Arm Movement.

All subjects completed all manual tasks. For the locomotion tasks, only subject 2 was able to complete the stair climbing task. She also completed the TUG task without any support. Subjects 1 and 3 were able to complete the TUG task using a walking frame. Additionally, they performed the TUG task with their wheelchair. Subject 4 completed the TUG task only with a wheelchair.

#### ***Estimation of motor skill rating***

We used a linear regression to estimate the motor capacity rating from the collected data. Ratings from the movement scientists served as ground truth.

Regression is a supervised machine learning method with the goal to predict a target variable *t* given a *D*-dimensional vector of input variables *x* (the features) [44]. The model estimates the output **y** as a linear function of the parameters **w** and **x**

$$y(x,w)=w_{0}+\overset{n}{\underset{j=1}{\sum }}w_{j}x_{j}.$$

*w*_0_ is the constant component, *x*_*j*_ are the values of the *j*-th feature at the different time instances (*n* features in total) and *w*_*j*_ is the *j*-th coefficient of the weight vector **w**. Note that vectors are represented in bold as opposed to scalars.

The squared error of the prediction *E*_*D*_(**w**) is then given by

$$E_{D}(w)=\frac{1}{2}{\left(t-w^{T}x\right)}^{2}$$

and is minimized by the algorithm.

Since features were not naturally on the same scale, we standardized features to range between 0 and 1. This standardization ensured that features were treated as equally important. Additionally, the target variables (i.e. the skill rating by the movement scientists) were standardized from 0 to 1 to achieve that target variables were in the same range for all activities and thus allowed for better comparison of the different estimation results.

For each task a separate regression model was trained. The model was trained on all data except that of a single assessment (one weekly session of one subject) and then tested on the remaining data, each model thus included data from all subjects. Note that this incorporated that there were twice as many data points for the single-handed tasks (data from the left and right hand were combined).

When a large number of features is used in a regression model, this yields “over-fitting”, which means that the estimation is very good, but it limits the generalization when including more data. Therefore, to avoid over-fitting, we included only significant features (*p* < 0.05) in the regression model. To obtain significance for features, the hypothesis of the corresponding weight *w*_*j*_ being equal to zero was tested using an Independent Samples T-Test.

To evaluate the quality of the estimation, we calculated the root mean squared error (RMSE) between the target variable and the prediction.

We chose to use the RMSE as an evaluation measure since it in general has the same unit as the data. Since the target data is standardized to a range from 0 to 1, this allows for an easy interpretation of the RMSE. As an additional evaluation criterion, we calculated the correlation coefficients between our regression estimate and the ground truth.*p*-values for testing the hypothesis of no correlation were also calculated.

## Results

**Figure 9:**
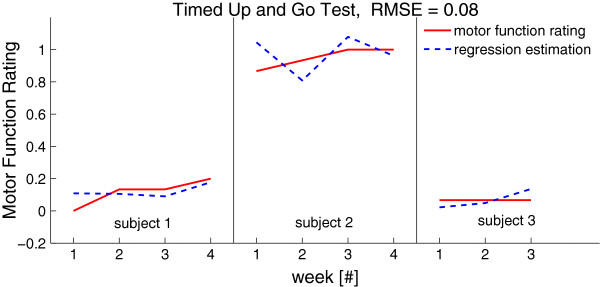
**Motor function rating by movement scientists and as estimated by our regression model.** Model includes data from the three subjects who were able to walk across the four weeks. The first two subjects increased their performance over the four weeks.

In this section, we present the results of the regression analysis described before with plots for each tasks individually. For better illustration, we exemplarily describe the approach with the data from the TUG test (see Figure 9).

Since only three of the four subjects were able to walk, there were only 11 data points available for this task. The movement scientists’ rating, which served as ground truth, and our estimation from the regression are depicted in Figure 9. The first four data points are from subject 1 walking with a walking aid along the four weeks, the next four data points of subject 2 who was able to walk on her own, and the last three of subject 3 walking with the help of a walking frame. It is observable that the first 2 subjects improved over the course of four weeks, which could also be obtained from the regression.

**Figure 10:**
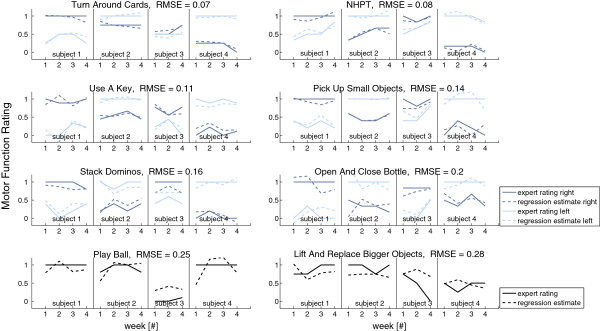
**Ground truth and regression estimation of the motor function using sensor data.** Only statistically significant features (*p*<0.05, *α*=0.05) were used for the estimation, see Table 4. Note that for the single-handed tasks the results for the left and right hand performing the task are depicted whereas for the bimanual tasks there is only one set of graphs. Subplots are ordered with decreasing RMSE. The best result was achieved for the Turn Around Cards and the NHPT tasks.

Figure 10 depicts the regression results for the remaining tasks ranked according to the RMSE. The correlation coefficients *r* between our regression estimate and the experts’ rating and the according *p*-values are listed in Table 3.

**Table 3:** **Correlation coefficients*r* and*p*-values to indicate the goodness of fit between our regression estimate and the experts’ motor performance rating**

**Task**	**Correlation coefficient**	***p*-value**
Turn around cards	0.97	<0.01
Pick up small objects	0.92	<0.01
Stack dominos	0.92	<0.01
Lift and replace bigger objects	0.43	<0.05
Open and close a bottle	0.86	<0.01
Use a key	0.95	<0.01
NHPT	0.96	<0.01
Play ball	0.75	<0.01
TUG	0.98	<0.01

Abbreviations: NHPT = Nine Hole Peg Test; TUG = Timed Up And Go.

**Table 4:** ***p*-values obtained for the different features and tasks from the regression model**

**Task**					**Feature**						
	**TIME**	**MI**	**MIV**	**DF**	**SM**	**ARE**	**RANG**	***T***_***step***_	$\frac{T_{\mathrm{stpR}}}{T_{\mathrm{stpL}}}$	***T***_***stance***_	$\frac{T_{\mathrm{sncR}}}{T_{\mathrm{sncL}}}$
Turn cards	**<0.01**	>0.05	>0.05	>0.05	>0.05	>0.05	**<0.01**				
Small objects in Bin	**<0.01**	>0.05	**<0.01**	**<0.01**	>0.05	>0.05	>0.05				
Stack dominos	**<0.01**	>0.05	>0.05	>0.05	**<0.01**	>0.05	>0.05				
Open/close bottle	**<0.01**	>0.05	>0.05	>0.05	>0.05	>0.05	**<0.01**				
Open door w/ key	**<0.01**	>0.05	>0.05	>0.05	>0.05	>0.05	**<0.01**				
NHPT	**<0.01**	>0.05	>0.05	**<0.01**	>0.05	**<0.05**	>0.05				
Replace larger objects (R)	**<0.05**	>0.05	>0.05	>0.05	>0.05	>0.05	>0.05				
Replace larger objects (L)	**<0.05**	>0.05	>0.05	>0.05	>0.05	>0.05	>0.05				
Play ball (R)	>0.05	>0.05	>0.05	>0.05	>0.05	**<0.01**	>0.05				
Play ball (L)	>0.05	>0.05	>0.05	>0.05	>0.05	**<0.01**	>0.05				
TUG	>0.05	>0.05	>0.05	>0.05	**<0.05**	>0.05	>0.05	**<0.01**	>0.05	>0.05	>0.05

Abbreviations: TIME = Task Completion Time, MI = Movement Intensity, MIV = Movement Intensity Variation, DF = Dominant Frequency, SM = Smoothness of Movement, ARE = Average Rotation Energy, RANG = Range of Angular Rotation, *T*_*step*_ = step duration, *T*_*stance*_ = stance duration. Significant features (*p*<0.05) are marked bold and resemble the features that were used in the regression model to estimate motor function of the individual task. Features for the tasks stair climbing and TUG (wheel chair) are not included since there were too few data and the target was constant, respectively (see text for details).

We achieved the best estimation of the upper extremity tasks for the NHPT. The significant features for this task were TIME, dominant frequency (DF), and average rotation energy (ARE), see Table 4. The worst estimation was obtained for the 2 two-handed tasks (play ball and replace larger objects). For all single-handed tasks we found RMSE ≤ 0.2.

For the TUG Test with a wheel chair subjects did not improve over the course of the four weeks and we were thus not able to draw a conclusion on which features were most important to predict motor function. Only one subject was able to walk the stairs independently, which did not provide sufficient data to fit the regression. However, from looking at the features, we assume these might still yield valuable information for motor function assessment.

The average RMSE across all tasks performed by the hands (i.e. the tasks depicted in Figure 10) was 0.16. Including the TUG test, we achieved a mean RMSE of 0.15. The *p*-values of the significance of the features are given in Table 4.

From the *p*-values of the features we found that TIME seemed to be the most important parameter describing motor function for the majority of tasks, which confirms our approach since for most tasks the goal was to perform the task as fast as possible. However, the dominant frequency (DF), the smoothness of the movement (SM), the average rotation energy (ARE), and the range of angular velocity (RANG) were also found to be significant for the assessment of some of the tasks.

### Towards a generalized approach

**Figure 11:**
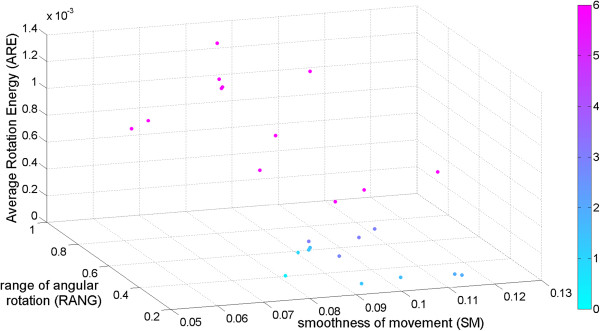
**Scatter plot of the three best task-independent features identified during the data analysis, namely average rotation energy (ARE), range of angular rotation (RANG), and smoothness of movement (SM).** Points indicate the feature values of the different subjects and weeks averaged over all tasks. Points are colored according to the performance rating of the expert. It can be observed that the points with better ratings have higher RANG, higher ARE, and higher SM. Since these values are the average over all performed tasks, this figure indicates the potential of these features for performance assessment in daily life when little or no information on the performed task is available.

To investigate the generalizability of the calculated features for motor function assessment, we calculated the mean values of features over all tasks performed with a single hand per subject and week. With this, we assumed we would not have had any information on the performed task. Features were chosen according to their significance as summarized by Table 4. TIME and DF were not included since they highly depended on the task performed. A scatter plot with the data points colored according to the summed function score of the appropriate week and subject is depicted in Figure 11.

It can be observed, that the scatter cloud moves in the feature space with increasing motor function.

## Discussion

In this work, we used a linear regression to estimate movement capacity from sensor data. Motor capacity ratings of experts served as ground truth. The deviation of our estimation and the experts’ rating was evaluated using the RMSE.

We found RMSE ≤ 0.2 for all single-handed tasks and 0.2 < RMSE < 0.3 for the bi-manual tasks, which might be explained by the amount of data available, since regression model estimates are usually better when more data points are entered [44]. However, this was in contrast with the RMSE of the TUG test, for which a low RMSE of 0.08 was achieved even though only 11 data points were available. We think this could be explained by the range of movement function ratings. For the TUG task, one subject was able to walk almost without visible impairment of gait. The other two subjects needed to use a walking frame and found it clearly challenging to walk 10 m. For the Play ball task and the Replace bigger objects task the differences between the different trials and subjects were not as notable.

When analyzing the significant features, we found that TIME was significant for most tasks, which was consistent with the tasks’ protocol since the state-of-the-art analysis of the performed tasks included task completion time extraction using a stopwatch and it was the goal for most tasks to complete them as fast as possible. However, time could be regarded as a capacity outcome rather than a capacity determining factor (cause and effect: fast performance due to secure and efficient performance). We wanted to investigate additional features that might indicate the differences between functional levels and serve more as capacity determining factor. For the other features, investigating dominant frequency (DF), smoothness of movement (SM), average rotation energy (ARE), range of angular velocity (RANG), and step duration (*T*_*step*_) seemed to be especially promising.

To investigate the generalizability across the different upper extremity tasks, we calculated the mean values of SM, ARE, and RANG across all single-handed tasks to simulate there would not have been knowledge on the task performed. When looking at a scatter plot of these features with data points colored according to the rating (summed over the 6 tasks), we found that the scatter moved in this feature space with the rating. This seemed to indicate that these features provide valuable information for motor function assessment even when no information is available on the task, which emphasizes our approach of going further out of the laboratory environment and monitoring the children in daily life, during which not much information on the task performed might be available. However, we are aware that daily life not necessarily includes the tasks performed in this study and that it remains to further investigation whether these features generalize for task performance assessment during daily living. Additionally, we plan to further investigate the generalizability on additional, different tasks that occur in daily life to validate this approach.

For a follow-up study in daily life only a minimal sensor setup should be used to maximize unobtrusiveness. We used the results of this study to learn about which sensor positions provide the most valuable information. We found that a minimal sensor setup of 3 sensors attached to the hip and both wrists would suffice to calculate the relevant features used in this work: The low signal amplitude on the upper arms yielded an exclusion of the upper arm sensor data prior to the feature calculation. Features were thus calculated from the remaining 8 sensors and fed into the model. In the model analysis we investigated, which features contributed significantly to the motor capacity estimate, see Table 4. These were features calculated from the wrists and the hip and the step duration, which was calculated from the feet sensors. However, the step duration can also be calculated from the hip sensor, yielding a sensor setup reduction to 3. Other parameters for which sensors at the feet would be needed such as stance duration were not found to contribute significantly to the motor capacity estimate.

## Limitations

Even though the results seem to be promising one would need to increase the number of participating children in order to validate the results and further investigate differences across different impairments. Increasing the number of activities would help with the validation of the generalizability of our approach towards daily life. Additional sensor modalities could be investigated for a more detailed and fine-grained analysis of the movements.

## Conclusion and outlook

We have presented the use of wearable sensors to assess motor function of children undergoing stationary rehabilitation using predefined tasks. With a regression we were able to estimate motor task capacity with an average RMSE of 0.15. The average correlation between experts’ ratings and our estimation was 0.86 (*p* < 0.05) across all tasks. We presented the calculation of features from the sensor data and identified important features for motor task performance assessment. Using a sensor setup of 10 sensors we investigated which sensors provided the most valuable information and were able to reduce the number of used sensors to three: one on each wrist and one on the hip. Envisioning our aim to perform data collection and analysis in daily living, a minimal sensor setup of three sensors would suffice to cover our desired areas of motor function assessment in a future study.

Envisioning our aim to perform data collection and analysis in daily living, three sensors would suffice to cover our daily life activities of interest and investigate the feasibility of assessing motor performance in the field.

## Endnotes

^a^ In machine learning and pattern recognition, a feature is a measurable heuristic describing a specific characteristic of data. Examples are mean value, standard deviation, or signal energy.

^b^ Dynamic Time Warping is an algorithm for measuring similarity between two sequences which may vary in time or speed.

^c^ 1 g$\hat{=}$ acceleration measured due to gravity, i.e. 1 g$\hat{=}$ 9.81 m/s^2^.

^d^ A movement with most energy concentrated around a dominant frequency can be described as smooth since this means that the movement is mainly performed at this frequency, i.e. not much movement is performed at different frequencies, which would indicate a more uncontrolled movement.

## Abbreviations

nfeatures: Number of features; r: Correlation coefficient; ARE: Average rotation energy; ArmSync: Synchrony of arm movement; CP: Cerebral palsy; DF: Dominant frequency; FAS: Functional ability score; FFT: Fast fourier transform; FT: Fourier transform; IMU: Inertial measurement unit; MI: Mean value of movement intensity; MIV: Movement intensity variation; NHPT: Nine hole peg test; RANG: Range of angular velocity; RMSE: Root mean squared error; SM: Smoothness of movement; TIME: Task completion time; TUG: Timed up and go test; WeeFIM: Functional independence measure for children; WMFT: Wolf motor function test; SWAB: Sliding window and bottom-up.

## Competing interests

The authors declare that they have no competing interests.

## Authors’ contributions

CS participated in the study design, performed the data analysis, and drafted the manuscript. RL participated in the study design, participated in the experiments, and helped to draft the manuscript. CG helped with the recruitment of subjects and participated in the experiments. HvH participated in the study design. BA participated in the study design and helped to draft the manuscript. GT participated in the study design and helped to draft the manuscript. All authors carefully reviewed the manuscript and approved its final version.

## Authors’ information

CS is doing her PhD in Electrical Engineering and Information Technology. Her research interest is in movement analysis using wearable technology. RL is a PostDoc in the field of pediatric rehabilitation. CG is doing her PhD in Movement Sciences. HvH is the Head of the Pediatric Rehab Research Group of the children’s rehabilitation hospital, Affoltern. BA is a PostDoc in the field of pervasive healthcare. GT is a professor in the field of Wearable Computing. His research interests include pervasive healthcare using wearable technology.
